# Innovative Non-Enzymatic Electrochemical Quantification of Cholesterol

**DOI:** 10.3390/s22030828

**Published:** 2022-01-22

**Authors:** Oana Elena Carp, Mariana Pinteala, Adina Arvinte

**Affiliations:** Centre of Advanced Research in Bionanoconjugates and Biopolymers, “Petru Poni” Institute of Macromolecular Chemistry, Grigore Ghica Voda Alley 41A, 700487 Iasi, Romania; rachita.oana@icmpp.ro (O.E.C.); pinteala@icmpp.ro (M.P.)

**Keywords:** cholesterol, electrooxidation, chronoamperometry, liebermann–burchard reaction

## Abstract

The use of the Liebermann–Burchard reaction in this study has been explored in the development of a simple, reliable, and robust quantitative electrochemical method to assay cholesterol, and hence provide a good alternative to colorimetric methods. The optimization of batch mode operation for electrochemical oxidation of cholesterol in the Liebermann–Burchard reagents included the applied potential and acidic volume. Tested using chronoamperometry, the developed method showed a high sensitivity (14.959 μA mM^−1^) and low detection limit (19.78 nM) over a 0.025–3 mM concentration range, with remarkable linearity (R^2^ = 0.999), proving an analytical performance either higher or comparable to most of the cholesterol sensors discussed in literature. The influence of possible interfering bioactive agents, namely, glucose, uric acid, ascorbic acid, KCl and NaCl, has been evaluated with no or negligible effects on the measurement of cholesterol. Our study was directed at finding a new approach to chemical processing arising from the use of external potential as an additional level of control for chemical reactions and the transfer of electrons between surfaces and molecules. Finally, the optimized method was successfully applied for the determination of cholesterol content in real blood samples.

## 1. Introduction

Cholesterol is an important lipid molecule in cell membranes and lipoproteins. Abnormal levels of cholesterol or its precursors have been observed in various human diseases, such as heart diseases, atherosclerosis, stroke, type II diabetes, brain diseases and many others. Therefore, accurate quantification of cholesterol is important for individuals who are at increased risk of these diseases. Ideally, the total cholesterol concentration in a healthy person’s blood should be less than 200 mg/dL (<5.17 mM). The borderline high is considered as 200–239 mg/dL (5.17–6.18 mM), and the high value is defined as above 240 mg/dL (≥6.21 mM). Analysis of cholesterol concentration in blood is a routine practice in medical screening or diagnosis and, therefore, a simple and practical detection of cholesterol is desirable and can be useful in the prevention and management of the cardiovascular disease.

Multiple analytical methods have been developed for analysis of cholesterol, including classical chemical methods [1,2], enzymatic assays [3,4], gas or liquid chromatography [5,6,7] and mass spectrometry [8,9,10]. Even though they perform satisfying for cholesterol detection, most of them are burdensome, time-consuming, require sample pre-treatment, a high-cost instrumental set-up, difficult standardization, and experienced personnel to operate. An electrochemical sensing approach overcomes these disadvantages and the worth of cholesterol (bio)sensors is already recognized and visible from the vast research in this field summarized in comprehensive reviews [11,12,13,14]. Most of the electrochemical assays for cholesterol are enzyme-based, where cholesterol oxidase (ChOx) is employed as the sensing elements and different electron mediators provide an appropriate potential gradient for electron transfer between the enzyme’s active site and electrode [13,15]. These biosensors exhibit the advantage of high selectivity and sensitivity, but they also have some disadvantages of requiring high technology for immobilizing enzymes on electrode surfaces, impaired enzymes, and limited reproducibility and life time. Therefore, finding a rapid and reliable method for cholesterol determination is still in demand and the development of non-enzymatic electrochemical sensors for measuring cholesterol over a wide linear concentration range, with high sensitivity and accuracy, is challenging [16,17,18,19,20].

**Scheme 1:**
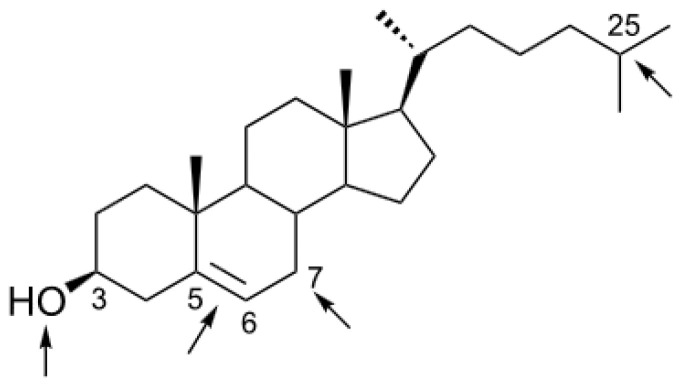
Favored sites for electrooxidation of cholesterol.

Although cholesterol has been regarded as an electrochemically inactive compound [21], recent studies [22,23] show that direct electrochemical oxidation of cholesterol is also possible and affords different products depending on the reaction condition. The first direct electrochemical oxidation of cholesterol was performed in glacial acetic acid on a platinum anode under constant current in a divided cell [22]. The reaction gave two major products, 7α-acetoxycholesterol and 7β-acetoxy-cholesterol, in a ratio of 10:3 and the voltammetric measurements indicated that cholesterol oxidation occurs at the allylic position (Scheme 1).

**Scheme 2:**
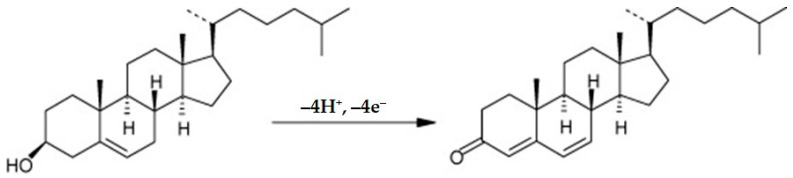
Expected reaction of the electrochemical oxidation of cholesterol.

Cholesterol has been shown to be electrochemically oxidized (Scheme 2) in acetonitrile containing LiClO_4_ at a carbon electrode to give cholesta-4,6-dien-3-one, under potentiostatic conditions at 1.9 V vs. Ag/AgCl electrode [24]. The product was formed through a four-electron, four-proton electrochemical process, but no explanation was given for the selectivity observed.

However, electrochemical biosensors for determining cholesterol have recently emerged to compete with the classic colorimetric assay involving Liebermann–Burchard (LB) reaction, where cholesterol is treated with sulfuric acid, acetic anhydride, and acetic acid provoking a blue color, due to the reaction of a hydroxyl group of cholesterol (Scheme 1). Although LB reaction is used extensively in many clinical laboratories, its major reaction pathway in acid has only been clarified recently [25]. However, the lack of specificity and color stability, the issue of temperature dependency, and the turbidity of a final color-developed solution have made colorimetric methods subject to significant concern regarding accuracy [26,27,28].

Generally, the behavior of cholesterol in strongly acidic solution is poorly understood and particularly, no information has been provided in literature with regard to the electrochemical oxidation of cholesterol in strong acidic media like those involved in the LB reaction.

In this work, we studied the reactivity of cholesterol under LB conditions, concomitantly applying a proper potential. It was proved that the LB reaction can be followed and amplified via the amperometric technique and can be used successfully to develop a simple, reliable, and robust quantitative amperometric method to assay cholesterol in serum samples, providing a good alternative to colorimetric methods.

## 2. Materials and Methods

### 2.1. Reagents

Cholesterol (≥99%), ascorbic acid (≥99%), uric acid (≥99%), sulfuric acid (98.08%), acetic anhydride (≥99%), chloroform (≥99.9%), glucose (≥99%), KCl (≥99%), NaCl (≥99%), tetrabutylammonium perchlorate (TBAP) (≥99%), ethanol (≥99.5%), methanol (≥99.9%), and HCl (37%) were obtained from Sigma-Aldrich. All other reagents used were of analytical grade. We used 50 mM TBAP dissolved in chloroform as a supporting electrolyte for electrochemical measurements. Cholesterol was dissolved in dry chloroform to prepare cholesterol solutions of different concentrations.

### 2.2. Instrumentation and Methods

A universal AutoLab PGSTAT 302N electrochemical system (Eco Chemie, Utrecht, The Netherlands) with a three-electrode cell was used. A glassy carbon electrode with renewable surface (3 mm diameter) was used as a working electrode, an Ag/AgCl reference electrode, and Pt wire counter electrode. The working electrode was polished with 0.05 and 0.3 µm alumina and abundantly rinsed with water and ethanol prior to each electrochemical measurement. Cyclic voltammetry was carried out to estimate the voltammetric profile of the cholesterol reaction and chronoamperometry to study the influence of applied potential on the electrochemical response of cholesterol and determination of cholesterol.

#### 2.2.1. Detection of Cholesterol in Solution

The reaction media was used according to reference [2] with some modification essential for electrochemical tests. In amperometric measurements, the baseline was recorded by applying the constant potential to the bulk reaction media consisting of the Liebermann–Burchard reagents: (1) 2 mL chloroform containing TBAP as electrolyte, noted as chloroform-TBAP; (2) 1 mL acetic anhydride; (3) 40 or 100 µL concentrated sulfuric acid. When the baseline current was stable (after approximately 100 s), 100 µL cholesterol solution was added in the cell and the oxidation current response was recorded. The added concentration of cholesterol was specified for each experiment.

#### 2.2.2. Detection of Cholesterol in Human Samples

Blood serum samples were collected by specialized personnel at “Prof. Dr. Nicolae Oblu” Emergency Clinical Hospital, Iasi, on the basis of interinstitutional scientific collaboration agreement and utilized after processing and cholesterol extraction. Analytical validation of the optimized method was accomplished using data from the Top Medical Grup laboratory (Iasi, Romania) in analyzing the same serum samples.

#### 2.2.3. Serum Processing

Blood collected in red-top (no additive) blood collection tubes (BD Vacutainer) was subjected to centrifugation at 5000 RCF for 5 min, followed by pipetting of the serum aliquots into cryovials and freezing at −80 °C. For cholesterol extraction, we used here a method adapted after (E.G Bligh and W.J. Dyer) [29]. The adjusted protocol was applied directly to 100 µL serum sample and the extraction was made using a 775 µL mixture of chloroform–methanol–water with a ratio of 1:1.6:0.5. For practical purposes, total lipid extraction was completed after adding 10 µL hydrochloric acid. Also, we tried extractions starting from 200 µL serum samples and the same volume of the chloroform–methanol–water mixture in order to evaluate the extraction efficiency. This concept was found to be very effective, and the miscible solvent mixture worked well as a lipid extractant, extracting the lipids from the non-lipids when chloroform and water were added.

### 2.3. Ethical Considerations

Informed consent was obtained from all subjects involved in the study. The processing of blood samples undergoing electrochemical analysis was in accordance with the European Directive EC No 206. The study protocol and all procedures included in the study were in accordance with the ethical standards within the Declaration of Helsinki.

## 3. Results and Discussions

### 3.1. Cyclic Voltammetry Preliminary Tests

**Figure 1:**
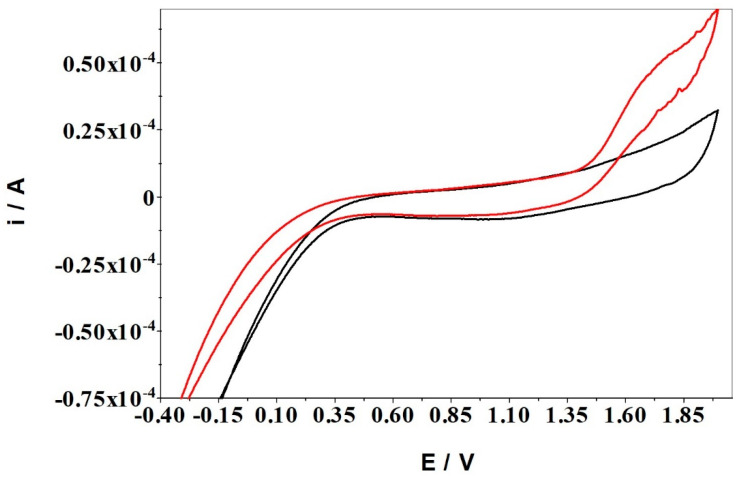
Cyclic voltammetry of 2 mL chloroform-TBAP + 1 mL acetic anhydride + 40 µL H_2_SO_4_ without cholesterol (black line) and with 0.64 mM cholesterol (red line), using GC and 0.1 V/s scan rate.

Figure 1 shows a representative current–potential curve obtained for cholesterol in a mixture of chloroform-TBAP, acetic anhydride and H_2_SO_4_ solution with a glassy carbon electrode. Cholesterol starts to oxidize at potentials more positive than 1.4 V vs. Ag/AgCl. As a control study, cyclic voltammetry was performed for the same reagents in the absence of cholesterol and no oxidation current was observed at around 1.4 V.

It is known that Lieberman–Burchard reagents used in colorimetric detection of cholesterol, gives a deep green color evolving in time [2,30]. This color begins as a purplish, pink color and progresses through to a light green then very dark green color. The color is due to the hydroxyl group (–OH) of cholesterol reacting with the reagents and increasing the conjugation of the unsaturation in the adjacent fused ring. In an electrochemical environment, when applying a potential, the reaction is greatly accelerated since the color changes instantly to green when the threshold of 1.4 V is reached and concomitantly, the oxidation current is increases greatly.

### 3.2. Amperometric Tests

**Figure 2:**
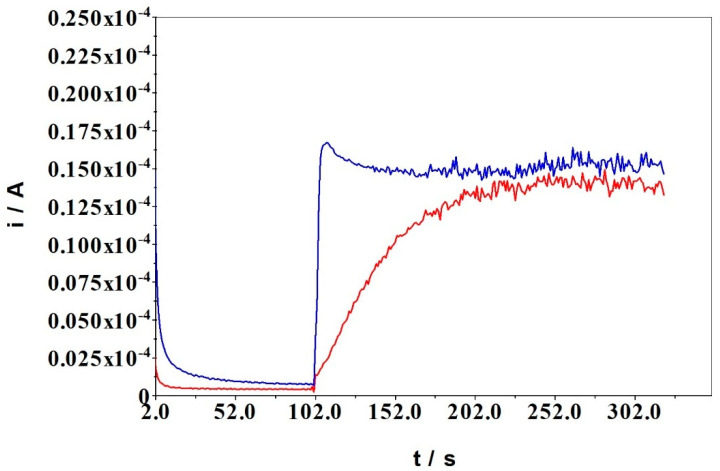
Amperometric response for: (blue line) 0.64 mM cholesterol added in the mixture (2 mL chloroform-TBAP + 1 mL acetic anhydride + 40 µL H_2_SO_4_) and (red line) 40 µL H_2_SO_4_ added in the mixture (2 mL chloroform-TBAP + 1 mL acetic anhydride + 0.64 mM cholesterol). Applied potential 1.5 V.

To assess the reliability of the electrochemical method, the oxidation response obtained for cholesterol in Lieberman–Burchard reagents was evaluated by amperometry in a stirred solution, applying 1.5 V, slightly higher than the onset potential observed in the CV data previously discussed. The baseline (i_0_) is recorded for (chloroform-TBAP + acetic anhydride + H_2_SO_4_) solution and when adding the cholesterol, the oxidation current increases rapidly (i_1_) (Figure 2, blue line) and the response (Δi_ox_) is depending on the cholesterol concentration. Since the Liebermann–Burchard reaction is a colorimetric assay [31,32,33], we concomitantly see the instant change of solution color.

Since the detection of cholesterol in these conditions involves more than an electrochemical mechanism, we explored the determination using a different approach, by adding the reagents in a different order: the cholesterol was already in the electrochemical cell together with chloroform-TBAP and acetic anhydride while recording the baseline and then, the H_2_SO_4_ was injected to the solution (Figure 2, red line). In this case, the oxidation current increased slowly, reaching the steady-state current after approximately 100 s, but the overall response (Δi_ox_) had the same value as in previous experiments. Correspondingly, the change of color was slower. The slow oxidation response indicated a sluggish electronic transfer, demonstrating that the electrochemical mechanism was preceded by a chemical one. The chemical reaction between acetic anhydride and sulfuric acid seemed to be of critical importance for the electrochemical reaction.

It is known that acetic anhydride and sulfuric acid reacted to give acetylsulfuric acid, which can be rearranged to sulfoacetic acid [31]:

$$H_{2}{\mathrm{SO}}_{4}+{\mathrm{Ac}}_{2}O\to \mathrm{AcOH}+{\mathrm{AcO}\text{-}\mathrm{SO}}_{2}\mathrm{OH}\to {\mathrm{HOSO}}_{2}{\text{-}\mathrm{CH}}_{2}\text{-}\mathrm{COOH}$$

**Figure 3:**
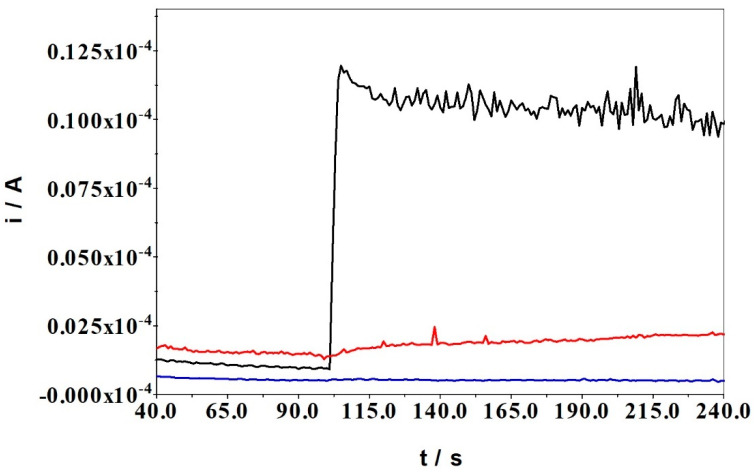
Amperometric response for: 0.64 mM cholesterol added in the mixture of: (black line) 2 mL chloroform-TBAP + 1 mL acetic anhydride + 40 µL H_2_SO_4_, (red line) 2 mL chloroform-TBAP + 40 µL H_2_SO_4_ and (blue line) 2 mL chloroform-TBAP + 1 mL acetic anhydride.

The sulfoacetic acid reacted with cholesterol, which was the first step in the derivatization of cholesterol, forming cholesta-diene [2,32,33], which was then involved in the electronic transfer with the electrode surface. This assumption was verified by an additional amperometric experiment in which the cholesterol was injected in the reaction mixture while omitting one reagent at a time (Figure 3). When the acetic anhydride was missing from the mixture, the addition of cholesterol induces only a small perturbation of amperometric current (0.4 µA), which indicated that H_2_SO_4_ was able to derivatize just a small part of cholesterol, which was then oxidized electrochemically. When the H_2_SO_4_ was missing from the mixture, the acetic anhydride was not able to induce any derivatization of cholesterol, consequently, none of it was undertaking oxidation, as proved by the lack of current response.

From the electrochemical results, we can conclude that conversion of sulfuric to acetylsulfuric acid was necessary to occur before introduction of cholesterol. Furthermore, in the presence of acetylsulfuric acid, cholesterol can variously undergo different pathways of transformation like acetylation, i-steroid formation, backbone rearrangement, dimerization, sulfonation, oxidation/desaturation, and aromatization [24,25]. Using the applied potential as a reagent for oxidation, a rapid electronic transfer is initiated between molecules obtained from a chemical reaction and electrode. The kinetics of electrochemical reaction may be depending on the applied potential or amount of acid.

### 3.3. Influence of Applied Potential

**Figure 4:**
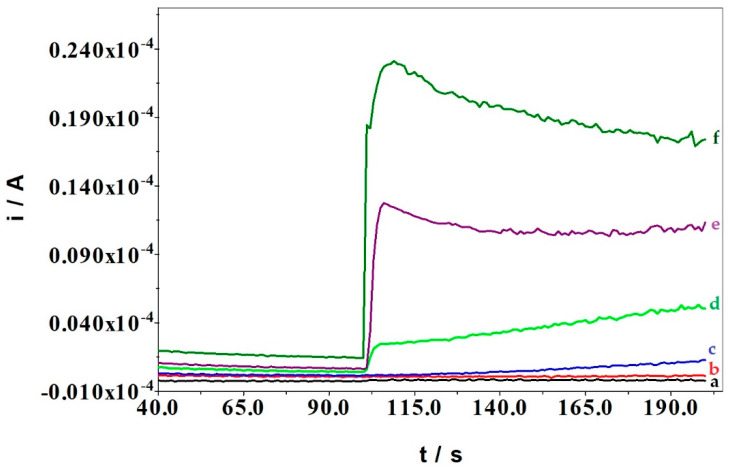
Amperometric response for 0.64 mM cholesterol added in the mixture (2 mL chloroform-TBAP + 1 mL acetic anhydride + 40 µL H_2_SO_4_) for different applied potentials: 0.8 V (**a**); 1.0 V (**b**); 1.2 V (**c**); 1.4 V (**d**); 1.5 V (**e**); 1.6 V (**f**).

The electrons and protons generated in electrochemical process are from the oxidation of organic material (cholesterol derivatives) present in the electrode vicinity and transferred to the electrode. Generally, the difference of potential between anode and cathode drives the oxidation or reduction reactions. In the present study, the potential applied in the electrochemical step was found to influence the cholesterol oxidation (Figure 4). Six different values of applied potentials 0.8, 1.0, 1.2, 1.4, 1.5 and 1.6 V were tested in the present study and the current response increased with the applied potential.

Because the current response was not very stable and the noise was quite significant at 1.6 V potential, we settled on 1.5 V to be used for further experiments. This value compared well with other applied potentials for direct electrooxidation of cholesterol reported in the literature such as 1.9 V [22,34] or 1.5 V [23].

### 3.4. Influence of Acid Concentration

**Figure 5:**
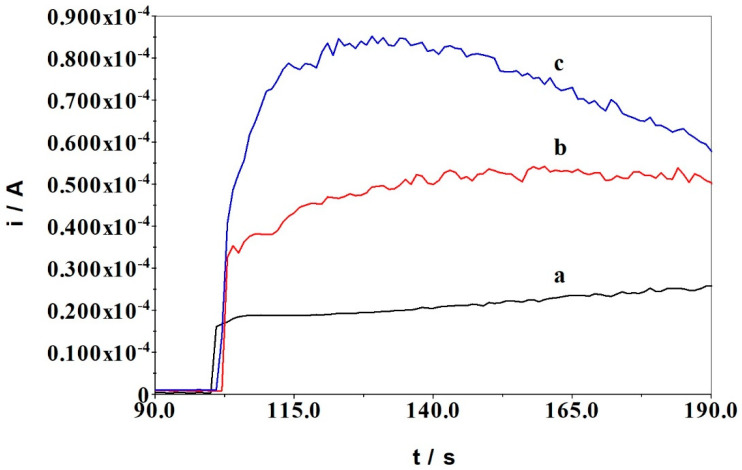
Amperometric response for 1 mM cholesterol added in the mixture (chloroform-TBAP + acetic anhydride) and different content of H_2_SO_4_: 40 µL (**a**); 100 µL (**b**) and 200 µL (**c**).

The influence of H_2_SO_4_ amount in the electrochemical response of cholesterol oxidation was studied using the optimal applied potential of 1.5 V. Figure 5 shows the anodic current obtained for 1 mM cholesterol oxidized in the LB mixture having a different amount of H_2_SO_4_.

It is obvious that the more acid involved in the reaction, the higher the response, but addition of 200 µL acid resulted in the instability of response current, probably due to the gradual degradation of products causing passivation of the electrode surface. The results are satisfactory for 100 µL of H_2_SO_4_ which was used in further experiments.

### 3.5. Calibration Curve

**Figure 6:**
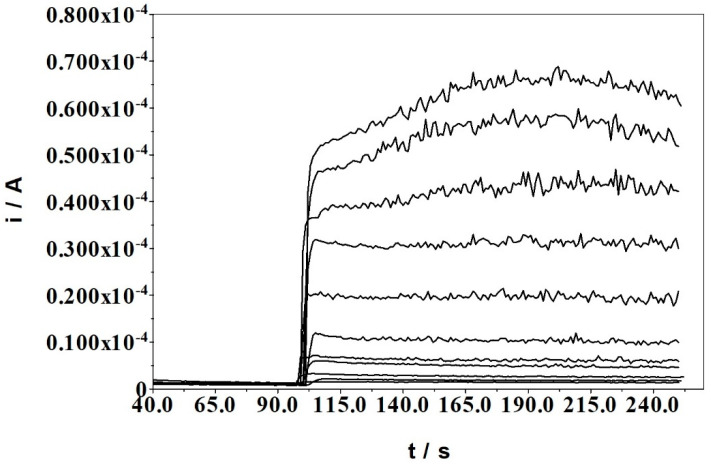
Amperometric response for increasing cholesterol concentrations (0.025, 0.05, 0.1, 0.2, 0.35, 0.5, 1, 2, 3, 5, 7 mM) added in the background mixture (chloroform-TBAP + 1 mL acetic anhydride + 100 µL H_2_SO_4_), applying 1.5 V potential.

**Figure 7:**
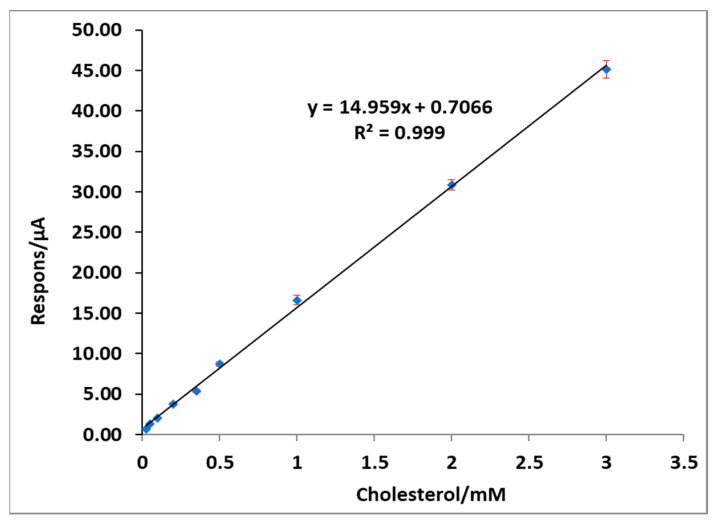
Corresponding calibration plot for cholesterol.

The sensitivity of the method and the linearity of response were evaluated by performing assays for cholesterol concentrations ranging from 0.025 to 7 mM, applying 1.5 V potential. The oxidation current response for these solutions increases with the concentration (Figure 6). From the amperometric data, a plot of current response (Δi) against cholesterol concentration was constructed (Figure 7). The developed method exhibited a linear relationship on a concentration range of 0.025–3 mM, with the highlighted equation. The calculated LOD for 3 σ/slope was 19.78 nM (σ—noise of the recorded current at zero concentration level).

### 3.6. Interferences

Many biological components in blood or physiological fluid such as ascorbic acid, uric acid, glucose, KCl and NaCl could be oxidized at the applied potential for the detection of cholesterol at the working electrode. This could cause interference masking or influencing the response current from the oxidation of cholesterol. For this reason, a series of selected compounds were tested individually and together with cholesterol by injecting them in the background mixture of chloroform-TBAP + acetic anhydride + H_2_SO_4_, under continuous stirring, applying the potential of 1.5 V. The concentration of tested compounds was higher than their normal content in serum.

**Figure 8:**
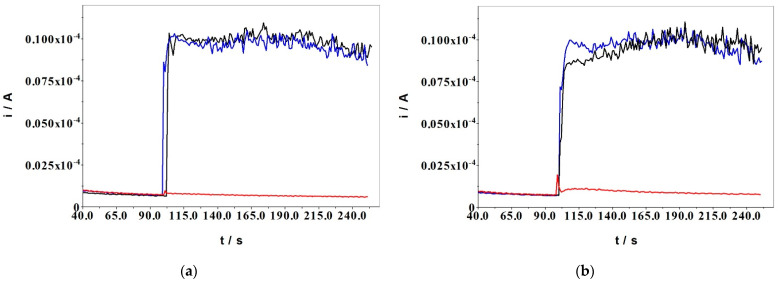
(**a**) Amperometric response for 0.64 mM cholesterol (black line), 0.2 mM Uric acid (red line) and Cholesterol + Uric acid (blue line) added in the mixture (chloroform-TBAP + acetic anhydride + H_2_SO_4_); (**b**) Amperometric response for 0.64 mM cholesterol (black line), 0.5 mM glucose (red line) and cholesterol + glucose (blue line) added in the mixture (chloroform-TBAP + acetic anhydride + H_2_SO_4_).

The amperometric responses for uric acid and glucose were recorded and depicted in Figure 8a,b), when they were injected either alone in the background mixture or together with cholesterol, previously mixed in a vial. According to the results, there was no interference effect on the cholesterol measurement.

**Figure 9:**
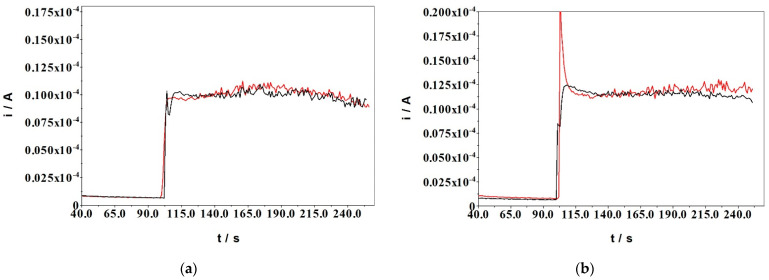
Amperometric response for 0.64 mM cholesterol injected in the reaction mixture (chloroform-TBAP + acetic anhydride + H_2_SO_4_) (black line) and in the reaction mixture containing also: (**a**) 0.5 mM KCl and (**b**) 0.1 mM ascorbic acid (red line).

Due to the insolubility of KCl in organic solvents, it was impossible for them to be injected as a solution in the reaction mixture. Therefore, KCl was added in the reaction mixture from the beginning, as an insoluble salt, while applying the potential. The baseline current was not affected, nor the response to cholesterol injected in the mixture, as shown in Figure 9a. The same approach was taken for ascorbic acid (Figure 9b) and no influence was observed in the response to cholesterol.

Taking into account the insolubility of NaCl in chloroform, we had to prepare its solution in methanol in order to test their possible interference in cholesterol measurement. For this reason, we tested prior to this the influence of methanol, since it is a polar solvent susceptible for electrooxidation at the applied potential.

**Figure 10:**
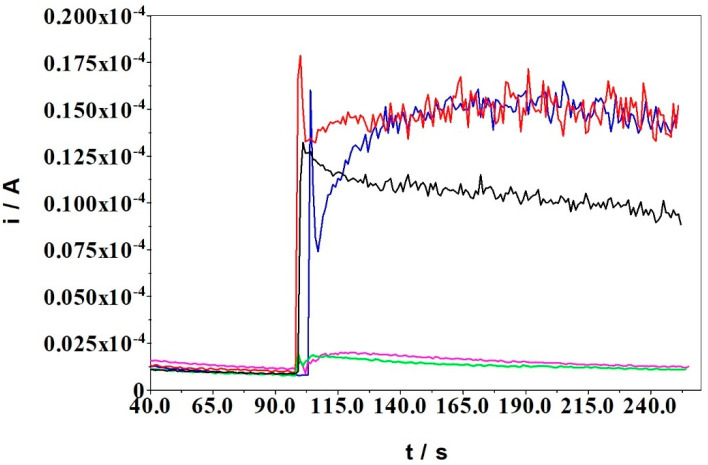
Amperometric response for 0.64 mM cholesterol (black line), 5 mM NaCl/methanol (green line), 105 µL methanol (pink line), NaCl/methanol + cholesterol (red line), and methanol + cholesterol (blue line) added in the background mixture (chloroform-TBAP + acetic anhydride + H_2_SO_4_).

When methanol was injected in the reaction medium (Figure 10, pink line), a small current response was observed due to its electrochemical oxidation, but this is not relevant for the studied analyte, since methanol is not normally found in blood or physiological fluids. The same response was obtained when NaCl/methanol solution was tested (Figure 10, green line) and the current is ascribed to the presence of methanol, as already proved. When methanol and NaCl/methanol (red and blue lines) were injected in the reaction medium together with cholesterol, a significant change of the current response was recorded compared to the response of pure cholesterol (black line). The increase of the oxidation current is again the effect of methanol electrooxidation.

### 3.7. Repeatability of the Method

The precision of the method was demonstrated by determining both intra-assay (repeatability) and inter-assay (intermediate) precisions (Table 1). The repeatability was proven by estimating the relative standard deviation (RSD) for 10 replicate determinations of 0.64 mM cholesterol.

The developed method was shown to be specific towards cholesterol, with a good sensitivity of determination, excellent linearity of response on a large range and low limit of detection. The method has been demonstrated to have a suitable level of precision and the analytical performance compares favorably to other reported studies in literatures for non0enzymatic electrochemical sensors for cholesterol, as emphasized in Table 2.

Combining the Lieberman–Burchard reaction with electrochemistry, the developed method overcomes some drawbacks existing for colorimetric methods or for enzymatic sensing platforms, such as time consumption, low sensitivity and selectivity, sophisticated instrumentation, standardization difficulties or limitations related to enzyme activity and stability. While the conventional colorimetric method needs at least 30 min to develop the color, the present electrochemical approach needs only few seconds to record the oxidation current response.

### 3.8. Detection of Cholesterol from Serum Samples

Blood samples were collected by specialized personnel at “Prof. Dr. Nicolae Oblu” Emergency Clinical Hospital, Iasi, and utilized after extraction in chloroform as explained in the experimental Section 2.2. Analytical validation of the electrochemical method was accomplished compared with data obtained from the Top Medical Grup laboratory (Iasi, Romania) analyzing serum samples from the same subjects involved in the study. The results shown in Table 3 demonstrate that cholesterol concentration in serum determined using the optimized electrochemical method agreed well with the data provided by the medical laboratory tests, revealing that the developed method was accurate. Moreover, the extraction method using 200 µL serum and 775 µL mixture of chloroform–methanol–water (1:1.6:0.5) was more efficient. The developed method was simple, fast and could be adapted to use in routine analyses.

## 4. Conclusions

Electrochemistry was successfully applied in combination with the Liebermann–Burchard reaction as an innovative and simple approach to determine cholesterol with high sensitivity and selectivity. Electrochemical reactions and procedures, when compared to other methods of analysis, are cheap, fast and environmentally friendly. Therefore, the cost of analysis could be more economical and practical.

It has been demonstrated that the Liebermann–Burchard reaction can be followed and amplified via the amperometric method and can be used successfully for analysis of cholesterol in serum samples. The developed method was shown to be specific towards cholesterol, with a good sensitivity of determination, excellent linearity of response on a large range, and low limit of detection. The optimized method serves as a reliable and robust alternative method to currently employed colorimetric or chromatographic methods which are more expensive or sophisticated. Substantial advantages over existing technology or methods, like simplicity, rapidity, specificity and good sensitivity, have been discussed.

## Figures and Tables

**Table 1:** Repeatability of cholesterol electrochemical determination.

	Assays	Mean Response Current (µA)	RSD
Intra-assay precision	triplicate consecutive determinations of the same concentration	10.56	1.97%
Inter-assay precision	10 determinations from different days	10.37	4.07%

**Table 2:** Analytical parameters of different non-enzymatic detection of cholesterol compared with proposed method in this work.

Electrode Modification	Detection Technique	Sensitivity	LOD	Linear Range	Ref.
Porous tubular AgNp/GCE	Amperometry, 0.35 V	-	1.8 × 10^−4^ M	2.8 × 10^−4^ M–3.3 × 10^−2^ M	[16]
Cu/Ni-carbon nanofiber/poly methyl orange	Chronoamperometry, 0.5 V	226.30 μA mM^−^^1^ cm^−2^	0.002 mg dL^−^^1^	0.04–600 mg dL^−^^1^	[17]
CuO-rGR/(1-methyl-3-octylimidazolium tetrafluoroborate)/CPE	Squarewave voltammetry	0.0902 μA μM^−1^	9 × 10^−9^ M	0.04–300.0 ×10^−6^ M	[18]
NiO/graphene/SiO_2_/Si	Amperometry	40.6 mA μM^−1^ cm^−2^	0.13 ×10^−6^ M	2–40 × 10^−6^ M	[19]
PtNP/(CNT)_24 bilayer_/ITO	Chronoamperometry, 0.7 V	8.7 μA mM^−1^ cm^−2^	2.8 × 10^−6^ M	0.005–10 ×10^−3^ M	[20]
Cu_2_O NPs/TNTs	Amperometry, −0.46 V	6034 μAmM^−1^ cm^−2^	0.05 × 10^−6^ M	24.4–622 ×10^−6^ M	[35]
Pt plate in NaClO_4_, KBr, DMF	Amperometry, 1.8 V	200 μAmM^−1^ cm^−2^	3.2 × 10^−6^ M	30–200 × 10^−6^ M	[36]
GCE in chloroform-TBAP, acetic anhydride, H_2_SO_4_	Amperometry, 1.5 V	14.959 μA mM^−1^	19.78 × 10^−9^ M	0.025–3 × 10^−3^ M	This work

**Table 3:** Serum sample analysis using the developed electrochemical method and compared to medical laboratory data.

Sample Number	Cholesterol Concentration (mg/dL)		Difference (A−B)/B×100
	Electrochemical Method (A)	Medical Lab Data (B)	
1a	140.68	156.1	−9.87
1b	150.13		−3.82
2a	295.69	318.2	−7.07
2b	308.94		−2.91
3a	146.76	160.4	−8.5
3b	153.17		−4.5
4a	204.51	221.3	−7.58
4b	208.64		−5.72
5a	247.06	273.4	−9.63
5b	273.99		0.21
6a	209.07	226.7	−7.77
6b	219.27		−3.27
7a	213.63	223.54	−8.11
7b	221.55		−5.92

a = sample obtained when 100 µL serum involved in the extraction. b = sample obtained when 200 µL serum involved in the extraction.
